# PERSONHOOD-BASED KNOWLEDGE: A NEW CONSTRUCT PREDICTING PERSONAL DEMENTIA FEAR

**DOI:** 10.1093/geroni/igz038.1745

**Published:** 2019-11-08

**Authors:** Alexandria R Ebert, Susan McFadden, Danica Kulibert

**Affiliations:** 1 West Virginia University, Morgantown, United States; 2 University of Wisconsin Oshkosh, Oshkosh, United States; 3 Tulane University, New Orleans, United States

## Abstract

Perhaps because the public is not well-versed on the biological and medical facts of dementia (biomedical knowledge; BK), or the life experiences and capabilities of persons living with dementia (personhood-based knowledge; PBK), dementia is one the most feared and stigmatized terminal illnesses (Alzheimer’s Society, 2007), typically resulting in social isolation (George, 2010). Similar to personal dementia fear (fear of developing dementia; PDF), dementia worry has been associated with suicide ideation (Cui et al., under review), which is a predictor of accepting attitudes toward physician-assisted suicide (Wolfgag, 2017). Findings from the development and testing of a conceptual model of social comfort indicated that people with higher levels of PBK had higher levels of social comfort towards persons with dementia (Ebert, Kulibert, & McFadden, 2019). The present study is a secondary analysis of data obtained from Wisconsin residents through an online platform and community outreach efforts (Ebert, Kulibert, and McFadden, 2019; N = 645) to examine whether individuals with higher levels of PBK have lower levels of PDF. A hierarchical linear regression revealed that PBK and BK were significant predictors of PDF (β = -.13, p < .05; β = .108, p < .05, respectively). However, when controlling for age (β = .14, p < .01) and knowing a friend or family member with dementia (β = -.19, p < .001), only PBK remained a significant predictor (β = -.16, p < .01). Results suggest that enhancing PBK through interactions with people living as well as possible with dementia could reduce PDF.

